# Rhus Toxicodendron in the Treatment of Enuresis

**Published:** 1885-09

**Authors:** 


### Rhus Toxicodendron in the Treatment of Enuresis.

Dr. G. W. Willeford, of Glendale, Ind., says that the fluid extract of rhus aromatica gives permanent relief in seventy-five per cent. of the cases treated, with great improvement in the remaining twenty-five per cent. It is the most efficient remedy yet tried by the observer for enuresis.—*Med. Record.*

